# Genomic Identification of Multidrug-Resistant *Salmonella* Virchow Monophasic Variant Causing Human Septic Arthritis

**DOI:** 10.3390/pathogens10050536

**Published:** 2021-04-29

**Authors:** Zhenyu Wang, Haiyan Xu, Chao Chu, Yuanyue Tang, Qiuchun Li, Xinan Jiao

**Affiliations:** 1 Key Laboratory of Prevention and Control of Biological Hazard Factors (Animal Origin) for Agri-Food Safety and Quality, Ministry of Agriculture of China, Yangzhou University, Wenhui East Road 48, Yangzhou 225009, China; DX120200156@yzu.edu.cn (Z.W.); MZ120191177@yzu.edu.cn (C.C.); tangyy@yzu.edu.cn (Y.T.); 2 Jiangsu Key Lab of Zoonosis/Jiangsu Co-Innovation Center for Prevention and Control of Important Animal Infectious Diseases and Zoonoses, Yangzhou University, Wenhui East Road 48, Yangzhou 225009, China; 3 Joint International Research Laboratory of Agriculture and Agri-Product Safety, Yangzhou University, Wenhui East Road 48, Yangzhou 225009, China; 4 Nantong Center for Disease Control and Prevention, Gongnong South Road 189, Nantong 226007, China; ntjk@ntcdc.cn

**Keywords:** *Salmonella* *enterica* serovar Virchow (*S.* Virchow), *Salmonella* 6,7,14:r:-, CRISPR typing, core genome MLST (cgMLST), *Salmonella* genomic island 2 (SGI2)

## Abstract

The monophasic variant of *Salmonella* Typhimurium has emerged and increased rapidly worldwide during the past two decades. The loss of genes encoding the second-phase flagella and the acquirement of the multi-drug resistance cassette are the main genomic characteristics of the *S.* Typhimurium monophasic variant. In this study, two *Salmonella* strains were isolated from the knee effusion and feces of a 4-year-old girl who presented with a case of septic arthritis and fever, respectively. Primary serovar identification did not detect the second-phase flagellar antigens of the strains using the classical slide agglutination test. Whole-genome sequencing analysis was performed to reveal that the replacement of the *fljAB* operon by a 4.8-kb cassette from *E. coli* caused the non-expression of phase-2 flagellar antigens of the strains, which were confirmed to be a novel *S.* Virchow monophasic variant (*Salmonella* 6,7,14:r:-) by core-genome multi-locus sequence typing (cgMLST). Compared to the 16 published *S.* Virchow genomes, the two strains shared a unique CRISPR type of VCT12, and showed a close genetic relationship to *S.* Virchow BCW_2814 and BCW_2815 strains, isolated from Denmark and China, respectively, based on cgMLST and CRISPR typing. Additionally, the acquisition of *Salmonella* genomic island 2 (SGI2) with an antimicrobial resistance gene cassette enabled the strains to be multidrug-resistant to chloramphenicol, tetracycline, trimethoprim, and sulfamethoxazole. The emergence of the multidrug-resistant *S.* Virchow monophasic variant revealed that whole-genome sequencing and CRISPR typing could be applied to identify the serovaraints of *Salmonella enterica* strains in the national *Salmonella* surveillance system.

## 1. Introduction

*Salmonella enterica* includes more than 2500 serovars and represents a major foodborne pathogen, which mainly causes gastroenteritis. However, focal suppurative infections of almost any organ may occur and produce different characteristic clinical syndromes [1,2]. Meningitis is diagnosed in less than 1% of clinical salmonellosis; *Salmonella* infections account for 0.8% of osteomyelitis cases. *Salmonella* infection also causes abscesses, including intra-abdominal infections, spontaneous peritonitis, splenic abscesses, and knee joint infections [3] Many non-typhoid *Salmonella* serovars, including *S.* Enteritidis, *S.* Typhimurium, *S.* Newport, *S.* Choleraesuis, *S.* Ohio, and *S.* Virchow, have been reported to be the causal factor in osteomyelitis or septic arthritis in humans [4,5,6,7,8,9,10]. Fever and back pain were reported as the main symptoms for vertebral osteomyelitis caused by *Salmonella* infection [5]. A case report showed that *S.* Ohio caused septic arthritis and a bone abscess in a 44-year-old immunocompetent man in Japan, and the *S.* Ohio strain was isolated from his bone abscess and from joint tissue of his swollen left knee [6]. *S*. Newport was also reported to cause left knee pain in a 36-year-old man with a fever of 38 °C and new-onset diabetes, whereas the bacteria were isolated from his blood, pus, knee aspirate, and tibia tissue [7]. *S.* Montevideo was another rare *Salmonella* serovar, identified to induce septic arthritis of the knee in a 59-year-old woman who presented with a painful and swollen right knee associated with a fever of 38.8 °C [8]. *S.* Virchow has also been connected with meningitis and knee septic arthritis [1,2,3,4,5,6,7,8,9,10].

Serotyping of *Salmonella* is performed by means of the classical slide agglutination test of bacteria with specific sera to identify the somatic (O) and flagellar (H) antigens based on the White–Kauffmann scheme [11]. With the development of CRISPR typing and whole-genome sequencing technologies, CRISPR-SeroSeq and genome-based *Salmonella* serotyping methods have become popular and effective [12,13]. In addition, CRISPR typing can differentiate strains belonging to the same serovar, whereas core-genome multi-locus sequence typing (cgMLST) can reveal the phylogenetic relationships of *Salmonella* strains from different sources [14,15].

This study reports the case of infection in the knee joint by an *S.* Virchow monophasic variant (*Salmonella* 6,7,14:r:-) in China for the first time. The novel serovariant without the *fljAB* operon was identified using both whole-genome sequencing analysis and the slide agglutination test. CRISPR typing and cgMLST were applied to demonstrate the phylogenetic relationship of the *S.* Virchow monophasic variant strains with the 16 published *S.* Virchow isolates. In addition, the antimicrobial resistance genotype and phenotype were determined in order to reveal the evolution of the monophasic variant strains.

## 2. Results and Discussion

### 2.1. Serovar Identification of Salmonella Strains Using the Slide Agglutination Test

According to the species identification by the VITEK-2^®^ Compact, *Salmonella* was isolated from both the knee effusion and stool samples, although not from the blood. Serovar identification showed that the two strains (YZU1797 and YZU1798) displayed antigenic formulae of 6,7,14:r:- using the slide agglutination test. The phase-2 flagellar antigens were not detected using the serovar identification kit. It was expected that the strains would be identified as a serovariant of *S.* Infantis (6,7,14:r:1,5) due to its predominant infection in infants and children [16]. According to the report, nearly 80% of patients infected with *S.* Infantis were less than 12 years of age, and these isolates were recovered from stools, urine, blood, or other biological fluids [16].

### 2.2. Identification of S. Virchow Monophasic Variant Based on Whole Genome Sequencing Analysis

**Figure 1:**
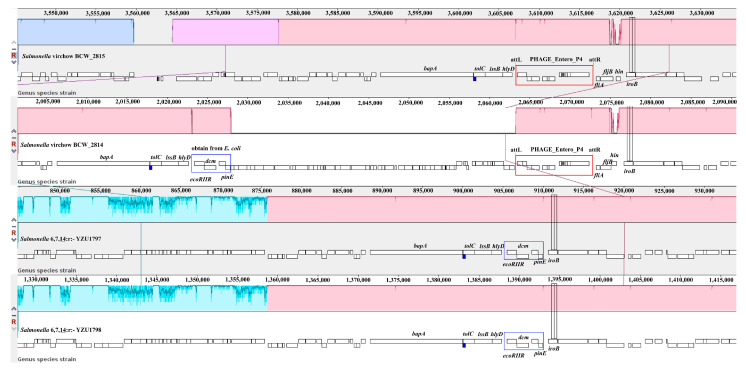
Sequence analysis of the *fljAB* operon in two *Salmonella* 6,7,14:r:- isolates. MAUVE software was used to compare the sequence of the *fljAB* operon and its neighboring regions in *Salmonella* 6,7,14:r:- with the corresponding sequences in *S.* Virchow BCW_2814 and BCW_2815. The red rectangle represents the phage Entero_P4 inserted in the site close to the *fljAB* operon, whereas the blue rectangle depicts a fragment acquired from *E. coli*.

Whole-genome sequencing analysis was subsequently performed to obtain the genome sequence of YZU1797 and YZU1798. The raw reads were then uploaded to the European Nucleotide Archive database under accession number PRJEB40529. Core genome MLST (cgMLST) was executed to reveal that both strains were *S.* Virchow (6,7,14:r:1,2). The *fljAB* operon (*fljA, fljB*, and *hin* genes) is involved in the synthesis of phase-2 flagellar antigens [17]. Homology analysis was then carried out on the *fljAB* operon and its surrounding sequences in YZU1797 and YZU1798 with the published *S.* Virchow genomes (Figure 1). The results showed that both strains lost the fragment of the *fljAB* operon, which was subsequently replaced by a ~4.8 kb fragment obtained from *E. coli*. With nomenclature similar to the *S.* Typhimurium monophasic variant (*Salmonella* 4,[5],12:i:-) [17], the serovar of both strains was named the *S.* Virchow monophasic variant (*Salmonella* 6,7,14:r:-). During the period from 2004 to 2009, *S.* Virchow ranked among the five most frequent serovars in humans, and the incidence of infected children under 5 years old was seven-fold higher than in those older than 5 years [18]. *S.* Virchow has also been confirmed to be one of the most common invasive serotypes infecting children, and can be isolated from the stool, blood, and synovial fluid [2]. However, this study is the first report of the *S.* Virchow monophasic variant causing infections in children, and comparative genomic analysis was therefore applied to reveal the origin of this variant if more strains were obtained from clinical cases.

### 2.3. Phylogenetic Relationship of S. Virchow and Its Monophasic Variant, Analyzed Using cgMLST and CRISPR Typing Methods

**Figure 2:**
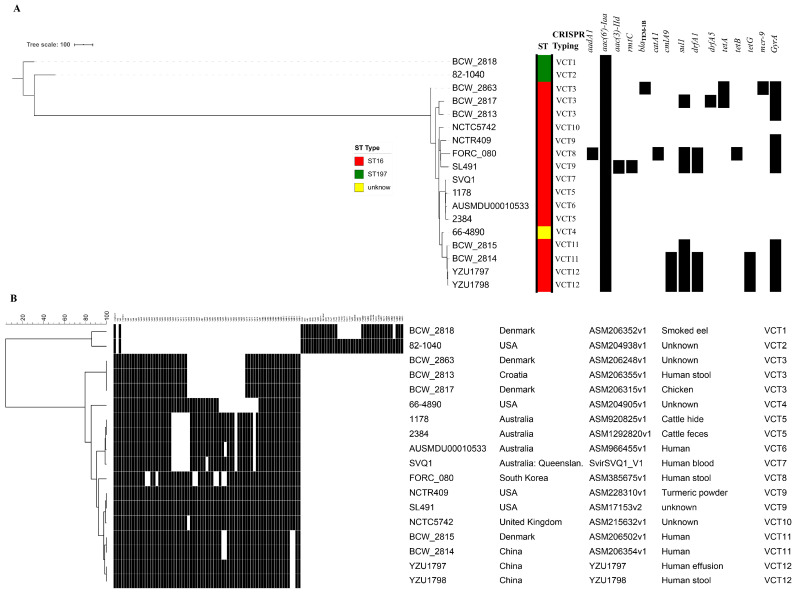
Phylogenic relationship of the two *Salmonella* 6,7,14:r:- isolates with 16 reported *S.* Virchow strains. (**A**) The phylogenic tree of 2 *Salmonella* 6,7,14:r:- isolates and 16 *S.* Virchow strains based on cgMLST analysis. The MLST type (ST), CRISPR type, and antimicrobial resistance genes are labeled on the right side with different colors and a black box, respectively. (**B**) CRISPR typing of 2 *Salmonella* 6,7,14:r:- isolates and 16 *S.* Virchow strains. The spacer names are shown on the upper side of the picture, with Vir + NO (VirN) for CRISPR 1 and VirB + NO (VirBN) for CRISPR 2, respectively. The black box represents the strain carrying the spacer. The maximum parsimony tree was constructed using BioNumerics version 7.5 software (Applied Maths, Sint-Martens-Latem, Belgium). The “*” represents the copy of the spacer Vir54 in the CRISPR array.

Sixteen published genomes of *S.* Virchow were downloaded and indexed to construct a phylogenic tree, using cgMLST analysis to reveal the genomic characteristics of both strains. Figure 2A demonstrates that the 18 strains were divided largely into two lineages. Two strains of ST197 belonged to lineage I, whereas the other 16 strains, including YZU1797 and YZU1798, belonged to lineage II. Both strains demonstrated a close relationship to strain BCW_2815 and BCW_2814 from Denmark and China, respectively (Figure 1 and Figure 2A). However, BCW_2815 and BCW_2814 preserved the *fljAB* operon with a prophage Entero_P4 inserted at the left side of the *fljA* gene (Figure 1), indicating their expression of phase-2 flagellar antigens.

CRISPR typing was also performed to demonstrate the serovar of both strains and the phylogenic relationship of the 16 strains (Figure 2B). Twelve *S.* Virchow CRISPR types (VCTs) were identified amongst the 18 strains, with VCT12 shared by YZU1797 and YZU1798. The majority of spacers identified in the two strains were VirN and VirBN (Figure 2B); both strains were identified as *S.* Virchow due to the spacer arrangements and their close relationship with the other *S.* Virchow strains of ST16 (Figure 2B), which is considered the major MLST type for *S.* Virchow [19]. Additionally, with similarities in the phylogenic tree based on cgMLST, CRISPR typing divided these 18 strains into two lineages (Figure 2B, Supplementary Figure S1). BCW_2818 and the 82-1040 strain of ST197, located in a separate lineage I, had unique VCT1 and VCT2 types (Figure 2B, Supplementary Figure S1). In lineage II, both Chinese strains of VCT12 showed a close relationship to BCW_2814 and BCW_2815 of VCT11, with a difference in two spacers (Figure 2B). The correspondence between CRISPR typing and cgMLST (Supplementary Figure S1) confirmed that CRISPR typing was able to be used as an efficient tool to analyze the phylogenic relationship of the isolates belonging to the same *Salmonella* serovar [20].

### 2.4. Antimicrobial Resistance Phenotypes and Genotypes 

**Figure 3:**
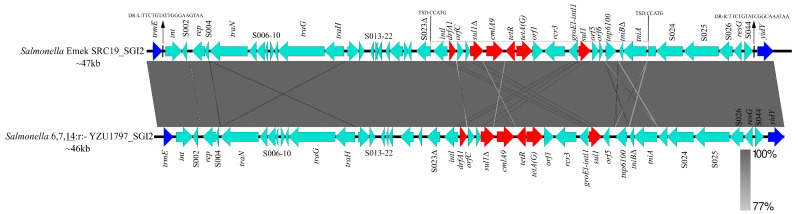
Homology analysis of SGI2 fragment between *Salmonella* 6,7,14:r:- and *S.* Emek. The red arrows represent the antimicrobial resistance genes, and the sky-blue arrows represent genes located in SGI2, whereas the dark-blue arrows show the genes at both sides of SGI2. The sequence between 2 TSD sites depicts the integron inserted into the SGI2 site through transposition.

The minimum inhibitory concentration (MIC) of the 12 antimicrobial agents for YZU1797 and YZU1798 showed that both strains were multidrug-resistant (MDR) to chloramphenicol, tetracycline, trimethoprim, sulfamethoxazole, and nalidixic acid (Table 1). Genome sequencing analysis revealed that the presence of *cmlA9*, *sul1*, *drfA1*, *tetA(G)*, and *gyrA*(S83F) in both isolates was involved in the antimicrobial resistance phenotype. Identification of antimicrobial resistance genes in the other 16 *S.* Virchow genes confirmed that BCW_2814 shared the same antimicrobial resistance genotype with YZU1797 and YZU1798 (Figure 2A). The antimicrobial resistance genes were located in the chromosome, since no plasmid was detected in either YZU1797 or YZU1798 by means of genome sequencing. Further analysis revealed that both *S.* Virchow monophasic variants acquired a ~43 kb *Salmonella* genomic island 2 (SGI2), including the antimicrobial resistance gene cassette (*drfA1-cmlA9-tetR-tetA(G)-sul1*) (Figure 3). The SGI2 was first reported in an *S.* Emek strain and was previously considered a variant from SGI1 (SGI1-J) [21]. The SGI2 or SGI1-J was also detected in three clinical *S.* Virchow isolates from human blood in 1993 and 1994 in China [22]. Within the SGI2, the integron carrying the antimicrobial resistance gene cassette was inserted in the S023 reading frame, flanked by a 5-bp target site duplication (TSD) (Figure 3), indicating that it was incorporated into the chromosome through transposition [23].

## 3. Materials and Methods

### 3.1. Sample Collection and Bacterial Isolation

The case of a 4-year-old girl with typhoid fever and knee arthritis is reported in this study. The patient was hospitalized in Nantong, Jiangsu, China. The patient presented a 39.5 °C fever and swelling of the knee joint, detected using nuclear magnetic resonance (NMR) imaging. The knee effusion, as well as stool and blood samples, were subjected to bacterial detection. Samples were rinsed in selenite broth medium and incubated at 37 °C for 16–24 h. The broth culture was then subcultured into MacConkey agar, blood agar, and XLT4 agar. The suspected colonies were subjected to species identification using a VITEK-2^®^ Compact (bioMerieux, Marcy-l’Etoile, France). The study was performed following the ethical guidelines of the 1975 Declaration of Helsinki and was approved by the Ethics Committee of the Chinese Centers for Disease Control and Prevention (CDC). Serovar identification of the strains was preformed using the *Salmonella* serovar identification kit (SSI, Denmark) according to the manufacture’s instructions.

### 3.2. Serovar Identification

The colonies on XLT4 agar (OXOID, Basingstoke, UK) plate suspected to be *Salmonella* were further selected and submitted for identification using the API 20E Test Kit (bioMérieux, Craponne, France). *Salmonella* isolates were serotyped by means of agglutination tests using the classical slide agglutination test of bacteria with specific sera to identify the somatic (O) and flagellar (H) antigens (SSI, Copenhagen, Denmark), based on the White–Kauffmann scheme. For phase switching analysis, bacterial swim media in Craigie tubes was prepared using Luria–Bertani (LB) broth, containing 0.3% agar, supplemented with anti-H:r (1:100 dilution) antiserum. Five colonies of *Salmonella* were inoculated in the tubes and incubated at 37 °C until motile bacteria were observed on the exterior of the tubing and/or the surface of the media. Bacterial suspensions taken from outside the inner tube were agglutinated with anti-H:r and anti-H:1 antiserum to identify the expression of phase 2 flagella [24].

### 3.3. Antimicrobial Susceptibility Test

The antimicrobial susceptibility testing was performed using the agar dilution method to determine the minimum inhibitory concentrations (MICs) of the isolates. Twelve antimicrobial agents were used, including kanamycin, streptomycin, amikacin, ampicillin, cefazolin, ampicillin + clavulanic acid, meropenem, chloramphenicol, trimethoprim/sulfamethoxazole, tetracycline, ciprofloxacin, and nalidixic acid. *E. coli* ATCC25922 was used as the quality control strain. The antimicrobial resistance results were interpreted according to the Clinical and Laboratory Standards Institute (CLSI) 2018 guidelines [25,26]. The experiment was performed in triplicate.

### 3.4. Whole Genome Sequencing Analysis

Genomic DNA of bacterial isolates was extracted using DNeasy Blood and Tissue Kits (Qiagen, Hilden, Germany), according to the manufacturer’s instructions. Qubit^®^ 3.0 fluorometer (Invitrogen, Carlsbad, CA, USA) was used to measure the DNA concentration. A total amount of 0.2 μg DNA was then used as the input material for the DNA library preparations using the NEB Next^®^ UltraTM DNA Library Prep Kit for Illumina (NEB, Ipswich, MA, USA), and subsequently sequenced using the 2 × 150 bp paired-end library on a HiSeq 2500 sequencing system (Illumina). Following trimming and filtering using the NGS_QC Toolkit (v2.3.3), the raw reads were subjected to de novo assembly by SPAdes 3.6 [27]. The subsequent annotation of the assembled genome was performed using Prokka version 1.12 [28]. Plasmids were identified using PlasmidFinder 2.1 (https://cge.cbs.dtu.dk/services/PlasmidFinder/, accessed on 11 February 2021). The multilocus sequence type of the strains YZU1797 and YZU1798 were obtained using the online tool MLST 2.0 (https://cge.cbs.dtu.dk/services/MLST/, accessed on 11 February 2021). The antimicrobial resistance genes and chromosomal mutations were analyzed using ResFinder 3.2 [29].

### 3.5. CRISPR Typing Analysis

The CRISPR-finder website (http://crispr.u-psud.fr/Server/, accessed on 11 February 2021) was used to obtain the components of the spacers in each strain. The Institute Pasteur CRISPR database (http://www.pasteur.fr/recherche/genopole/PF8/crispr/CRISPRDB.html, accessed on 11 February 2021) was used to obtain the name of each spacer in CRISPR1 and CRISPR2. The maximum parsimony tree based on the combined binary distribution patterns of all isolates was constructed using BioNumericus version 7.5 (Applied Maths, Sint-Martens-Latem, Belgium), as previously described [30].

## 4. Conclusions

We report a novel clinical *S.* Virchow monophasic variant (*Salmonella* 6,7,14:r:-), resulting in an infection in the knee joint of a 4-year-old girl. Replacement of the *fljAB* operon by a cassette from *E. coli* leads to the lack of phase-2 flagellar antigens in *Salmonella* 6,7,14:r:-. CRISPR typing and cgMLST revealed that the *S.* Virchow monophasic variant isolates (YZU1797 and YZU1798) were closely related to the previously reported human isolate from China, *S.* Virchow BCW_2814. Compared with the 16 published *S.* Virchow genomes, both strains obtained a unique ~43 kb SGI-2 fragment, including *cmlA9*, *sul1*, *drfA1*, and *tetA(G)* genes, providing multidrug resistance to chloramphenicol, tetracycline, trimethoprim, and sulfamethoxazole. Therefore, with the emergence of the MDR *S.* Virchow monophasic variant causing human salmonellosis, the identification of *Salmonella* serovars by whole genome sequencing analysis is recommended.

## Figures and Tables

**Table 1:** The antimicrobial resistance phenotype and genotype of *Salmonella* 6,7,14:r:- strains.

Classes	Antibiotics	YZU1797	YZU1798	Antibiotic Resistance Genes
Aminoglycoside	Kanamycin	S *	S	*aac(6′)-Iaa*
	Streptomycin	S	S	
	Amikacin	S	S	
Beta-lactam	Ampicillin	S	S	
	Cefazolin	S	S	
	Ampicillin + Clavulanic acid	S	S	
Carbapenems	Meropenem	S	S	
Phenicol	Chloramphenicol	R	R	*cmlA9*
Trimethoprim/Sulfonamide	Trimethoprim/Sulfamethoxazole	R	R	*sul1*, *drfA1*
Tetracycline	Tetracycline	R	R	*tetA(G)*
Fluoroquinolones	Ciprofloxacin	S	S	*gyrA*(S83F)
	Nalidixic acid	R	R	

* S represents sensitive, and R represents resistant.

## Data Availability

The raw reads of the whole genome sequencing project have been deposited in the European Nucleotide Archive database under accession number PRJEB40529.

## Supplementary Materials

The following are available online at https://www.mdpi.com/article/10.3390/pathogens10050536/s1. Figure S1: Comparison of the phylogenic trees constructed by cgMLST and CRISPR typing of the 18 *S.* Virchow strains and its monophasic variants.
