# Supplementary material for: Genomic Identification of Multidrug-Resistant Salmonella Virchow Monophasic Variant Causing Human Septic Arthritis
Source: Pathogens. 2021 Apr 29;10(5):536. doi: 10.3390/pathogens10050536 (PMC8146543; doi:10.3390/pathogens10050536)
Supplement: Supplementary file 1 [file pathogens-10-00536-s001.zip › Supplementary Figure.pdf]

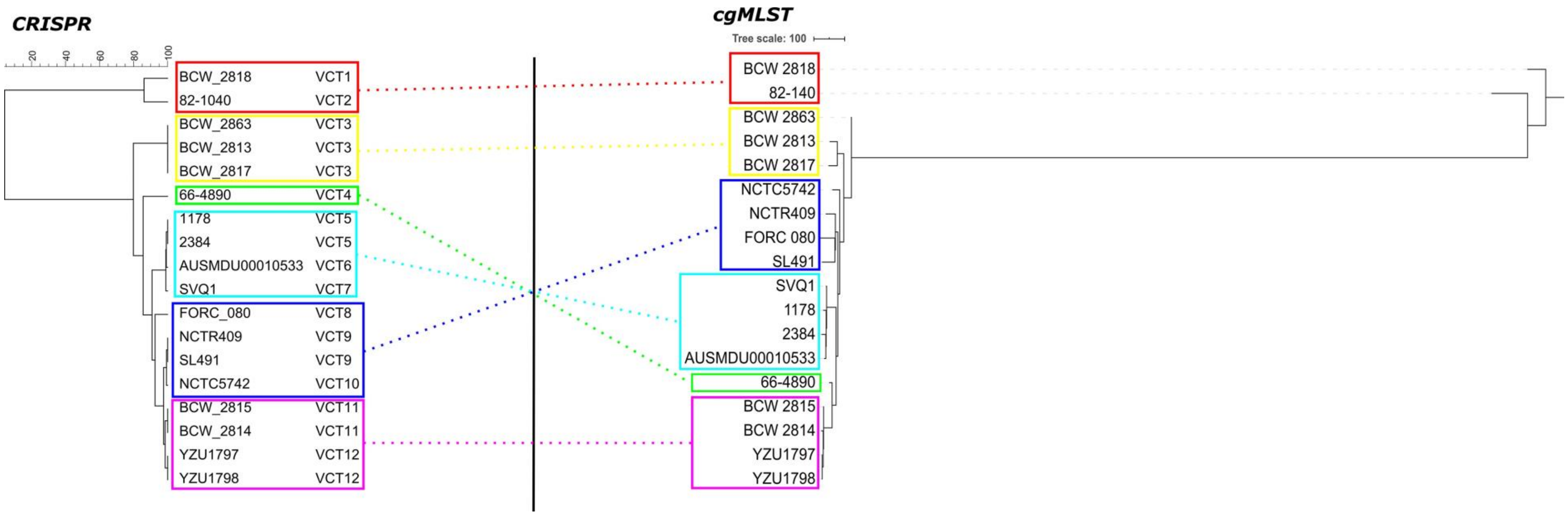

Supplementary Figure S1: Comparison of the phylogenic trees constructed by cgMLST and CRISPR typing of 18 strains. The rectangles with same colors represent the correspondent cluster between cgMLST and CRISPR typing analysis.
